# The Prevalence and Severity of Pain in Patients With Systemic Sclerosis

**DOI:** 10.1111/1346-8138.17843

**Published:** 2025-07-03

**Authors:** Sei‐ichiro Motegi, Mona Uchida‐Yamada, Yoshihito Shima, Taku Shimada, Haruka Ishii, Yoshito Ohya, Yasumasa Kanai

**Affiliations:** ^1^ Department of Dermatology Gunma University Graduate School of Medicine Maebashi Japan; ^2^ Medical Affairs Kyowa Kirin Co., Ltd. Tokyo Japan; ^3^ Laboratory of Thermo‐Therapeutics for Vascular Dysfunction Osaka University Graduate School of Medicine Suita Japan

**Keywords:** gap analysis, physicians, questionnaire, SF‐MPQ‐2, systemic sclerosis

## Abstract

We investigated pain prevalence and severity in systemic sclerosis (SSc) and patient–physician perceptions. This was an internet‐based survey that compared perceptions of pain type, location, severity, associated factors between patients with SSc and physicians, and pain treatment prescription patterns in Japan in March 2023. Data from 301 patients and 129 physicians revealed that 96.0% of patients experienced pain compared with 43.4% estimated by physicians. The median (interquartile range) Short‐form McGill Pain Questionnaire (SF‐MPQ‐2) pain score was 47.0 (14.0–88.0). Continuous pain had the highest score (16.0 [3.0–27.0]), followed by neuropathic pain (14.0 [5.0–25.0]), intermittent pain (11.0 [1.0–25.0]), and affective descriptors (5.0 [1.0–14.0]). Pain at joints, fingertips, Raynaud's phenomenon (RP), and skin tightening were most prevalent across multiple pain types. Pain at fingertips and RP‐affected locations were more common in limited cutaneous SSc (lcSSc) than in diffuse cutaneous SSc (dcSSc), and skin tightening was more common in dcSSc. Patients with dcSSc had significantly more severe pain than patients with lcSSc. Patients with nausea, insomnia, or diarrhea showed higher SF‐MPQ‐2 scores. Of the 129 physicians surveyed, 58.9% prescribed painkillers, 48.8% suggested self‐care, 42.6% treated skin symptoms, and 16.3% referred patients to pain clinics for further management. Compared to the percentage of patients having pain in each location, physicians tend to be less aware of pain in the muscles, head, and abdomen. Most patients with SSc experience pain, which physicians tended to underestimate. Physicians' awareness of patients' experiences should be improved to provide adequate treatment for pain in SSc.

**Trial Registration:** UMIN000050368

## 1 Introduction

Systemic sclerosis (SSc), also referred to as scleroderma, is an immune‐mediated multisystemic, rheumatic, and connective tissue disease with a high mortality rate [1]. It is characterized by fibrosis of the skin and internal organs [2, 3]. Circulating autoantibodies play an essential role in the pathophysiology of SSc and are a key feature of the disease [4]. SSc is categorized into diffuse cutaneous SSc (dcSSc) and limited cutaneous SSc (lcSSc) subtypes. dcSSc is characterized by skin tightening close to the trunk and higher disease activity and is more likely to be associated with rapidly progressive and fatal visceral organ involvement [5]. dcSSc is more common in anti‐topoisomerase I antibody‐positive or anti‐RNA polymerase (RNAP) III antibody‐positive patients. In contrast, lcSSc is associated with skin tightening limited to the periphery, milder disease activity, and is more common in patients with anti‐centromere antibodies [6, 7]. The prediction of future comorbidities using disease type and autoantibody information is becoming more accurate. However, there are many symptoms, such as pain and malaise, for which the originating complications are not thoroughly understood. Deepening our understanding of these symptoms can lead to the early detection of complications and appropriate treatment.

Pain is the most common and debilitating symptom of SSc, affecting over 80% of patients [8, 9, 10, 11]. More than a third of these patients describe their pain as moderate or severe [12]. Joint pain is reported to be one of the most common types of pain in SSc, with other symptoms associated with pain including Raynaud's phenomenon (RP), finger and hand ulcers, gastrointestinal symptoms, calcinosis, and polyneuritis/neuropathy [8, 10, 13, 14, 15, 16, 17, 18, 19]. Reportedly, greater pain severity is associated with longer disease duration and older patient age [8, 20]. Additionally, one report showed that pain scores did not differ between dcSSc and lcSSc [10], while other studies have reported that pain is associated with outcomes and disease severity [21, 22]. Pain has a negative effect on patients' quality of life (QoL) [19, 21, 23, 24, 25] and has been identified as the strongest predictor of disability and poor health‐related QoL in SSc [25].

Although the impact of pain in patients with SSc is gradually disclosed, few studies have quantified pain frequency, type, and severity in patients with SSc [9, 26]. The relationships between pain scores and subtypes of SSc, location, and symptoms have yet to be comprehensively assessed. It is also unclear whether there are differences between these patients' pain experiences and physicians' perceptions of what they experience. To manage pain appropriately, it is necessary to understand the characteristics of painful conditions. This study aimed to investigate the prevalence and severity of pain in patients with SSc and to explore gaps in physician and patient perceptions. In a previous report of this survey, we clarified the patient‐physician gaps in disease perception [27], and here, we focus on understanding the pain associated with SSc.

## 2 Methods

### 2.1 Study Design, Setting, Recruitment, and Data Collection

The study was a web‐based questionnaire survey conducted for physicians from March 14 to 17, 2023, and for patients from March 20 to 31, 2023. Members of the Patients' Association for Collagen Vascular Diseases Japan who were registered as having SSc were invited to participate via leaflets with the study web address and a notice about the study posted on the association's website. Announcements of recruitment for the study were also made within the Systemic Sclerosis Patient Community on the “LINE Open Chat”, an online social platform within the LINE mobile messaging application by LY Corporation (Tokyo, Japan), managed by QLife Inc. (Tokyo, Japan). Patients who were subscribed to the QLife newsletter were also notified about the study and directed to the study website. To prevent any inflow of non‐members to the website, a password for accessing links was included in the notice of this survey. Physicians who were members of the m3.com website, Japan's largest portal site specializing in medical care (https://corporate.m3.com/en), received the questionnaire survey.

Informed consent was obtained from patients through the website dedicated to this survey. If the user was a patient's family member, the user needed to consent to complete the questionnaire after obtaining the patient's consent. The study adhered to the Declaration of Helsinki and the Ethical Guidelines for Medical Research Involving Human Subjects. This study protocol was approved by the Research Ethical Review Committee of Kyowa Kirin Co. Ltd. under the approval number EC_0105.

### 2.2 Patients and Physicians

Patients ≥ 18 years of age with a diagnosis of SSc were included in this study. Patients were excluded if they did not know the Recipient Certificates issued for Specific Disease Treatment defined by the Act on Medical Care for Patients with Intractable Disease (Act No. 50 of 2014) in Japan [28]. Physicians could be included if they worked in hospitals with at least 20 beds and treated at least three patients with SSc per month.

### 2.3 Data Collection

The data collected in the questionnaire included the demographic and background characteristics of patients, pain treatment prescriptions, and the institutional department for physicians.

### 2.4 Study Outcomes

The main study outcomes were pain severity as graded on the Short‐form McGill Pain Questionnaire (SF‐MPQ‐2; Mapi Research Trust [Lyon, France]) https://eprovide.mapi‐trust.org/ [29, 30], with scores from 0 (no pain) to 10 (most severe pain) for each question and 0 to 220 for total scores and factors associated with pain. Pain location was analyzed along with the type of pain, that is, continuous, intermittent, and neuropathic pain, or affective descriptors. Additionally, the subtypes of dcSSc and lcSSc (based on questions regarding the extent of skin tightening), types of medications used, physicians' departments, and antibody status were assessed. The associations between symptoms and pain locations were assessed by combining the expected frequencies of these factors.

### 2.5 Statistical Analysis

This analysis was exploratory; thus, the sample size was determined based on feasibility and was not calculated for statistical hypothesis testing. The target was to collect 500 questionnaires, 300 from patients and 200 from physicians, as this number of patients and physicians could withstand stratified analysis.

The average of each of the specific pain classification questions (six each for continuous, intermittent, and neuropathic, and four affective questions) among the 22 questions in the SF‐MPQ‐2 was calculated for each patient, and the median score was calculated as a modified SF‐MPQ‐2 score by pain type. Sensory pain includes continuous, intermittent, and neuropathic pain, while affective descriptors refer to the affective perceptions of pain, such as tiring/exhausting, sickening, worrying, or punishing/cruel. The classification of pain per question followed previous reports [29, 30, 31]. Summary statistics such as median and interquartile (Q1–Q3) range were calculated for each pain type and SF‐MPQ‐2 total scores. Patients who answered with at least 1 point on the SF‐MPQ‐2 were considered “in pain”. Cluster analysis was performed to investigate the relationships between SSc symptoms, pain location, and patient background factors. To analyze the relationship between headache and medications for the treatment of RP, crude odds ratios and 95% confidence intervals were calculated for each dichotomized factor.

Data were analyzed using SAS (version 9.4; SAS Institute Inc., Cary, NC, USA) and the PROC VARCLUS procedure in SAS. Missing data were not imputed. The Wilcoxon rank sum test was used with a significance level of *p* < 0.05 (two‐tailed) to compare SF‐MPQ‐2 total scores for dcSSc and lcSSc.

## 3 Results

### 3.1 Responder Characteristics

Data from 301 patients and 129 physicians who completed the questionnaire were analyzed. The background characteristics of the patients (*n* = 301) surveyed have been previously reported [27]. Briefly, 216 patients (71.8%) responded directly, with the balance provided by patients' representatives, and 63.8% (192 patients) were female. Most patients (264 patients, 87.7%) were aged 30–80 years, with higher patient proportions in the ranges of 51–60 years (83 patients [27.6%]). In total, 144 patients (37.9%) had a disease duration of > 5 years, followed by 61 patients (20.3%) with 1–3‐year and 46 patients (15.3%) with 3–5‐year durations. There were similar numbers of patients with lcSSc and dcSSc (143 [47.5%] and 135 [44.9%], respectively), and 23 patients (7.6%) had unknown status. The most common departments in which physicians (*n* = 129) worked were rheumatology (66 physicians [51.2%]), followed by dermatology (27 physicians [20.9%]) and respirology departments (24 physicians [18.6%]).

### 3.2 Study Outcomes

**FIGURE 1:**
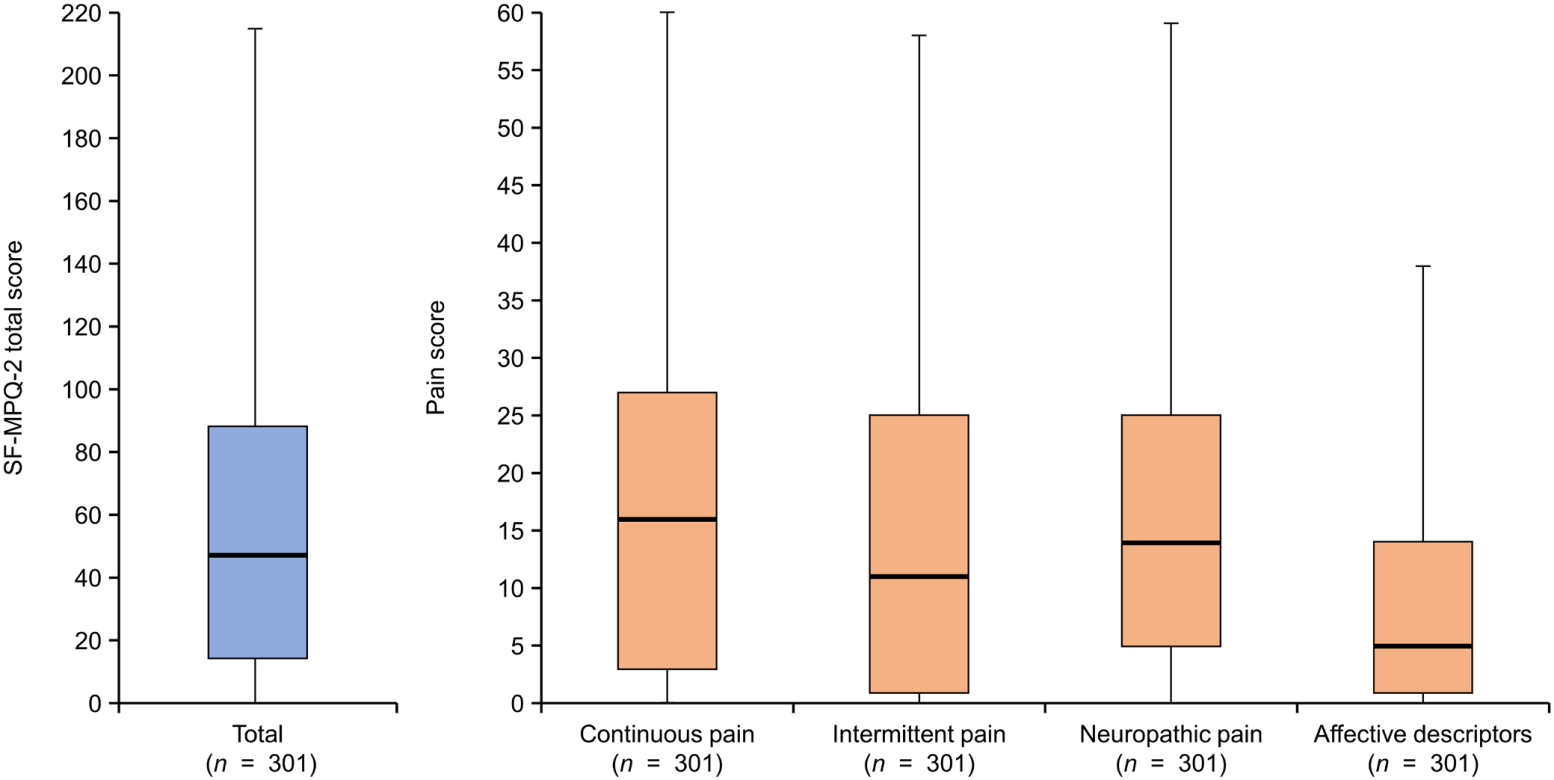
Median pain score by type of pain. Continuous, intermittent, and neuropathic types of pain are on a 60‐point scale, and affective descriptors are on a 40‐point scale. The total score of each of the four types of SF‐MPQ‐2 was calculated. The box represents quartiles 1–3, the line in the box shows the median value, and the upper and lower limits of the whiskers show the maximum and minimum values, respectively. SF‐MPQ‐2, Short‐form McGill Pain Questionnaire.

The prevalence of pain in patients with SSc was 96.0%. The median (Q1–Q3) SF‐MPQ‐2 total score was 47.0 (14.0–88.0) and was numerically the highest for continuous pain at 16.0 (3.0–27.0), compared with 11.0 (1.0–25.0) for intermittent pain, 14.0 (5.0–25.0) for neuropathic pain, and 5.0 (1.0–14.0) for affective descriptors (Figure 1).

**FIGURE 2:**
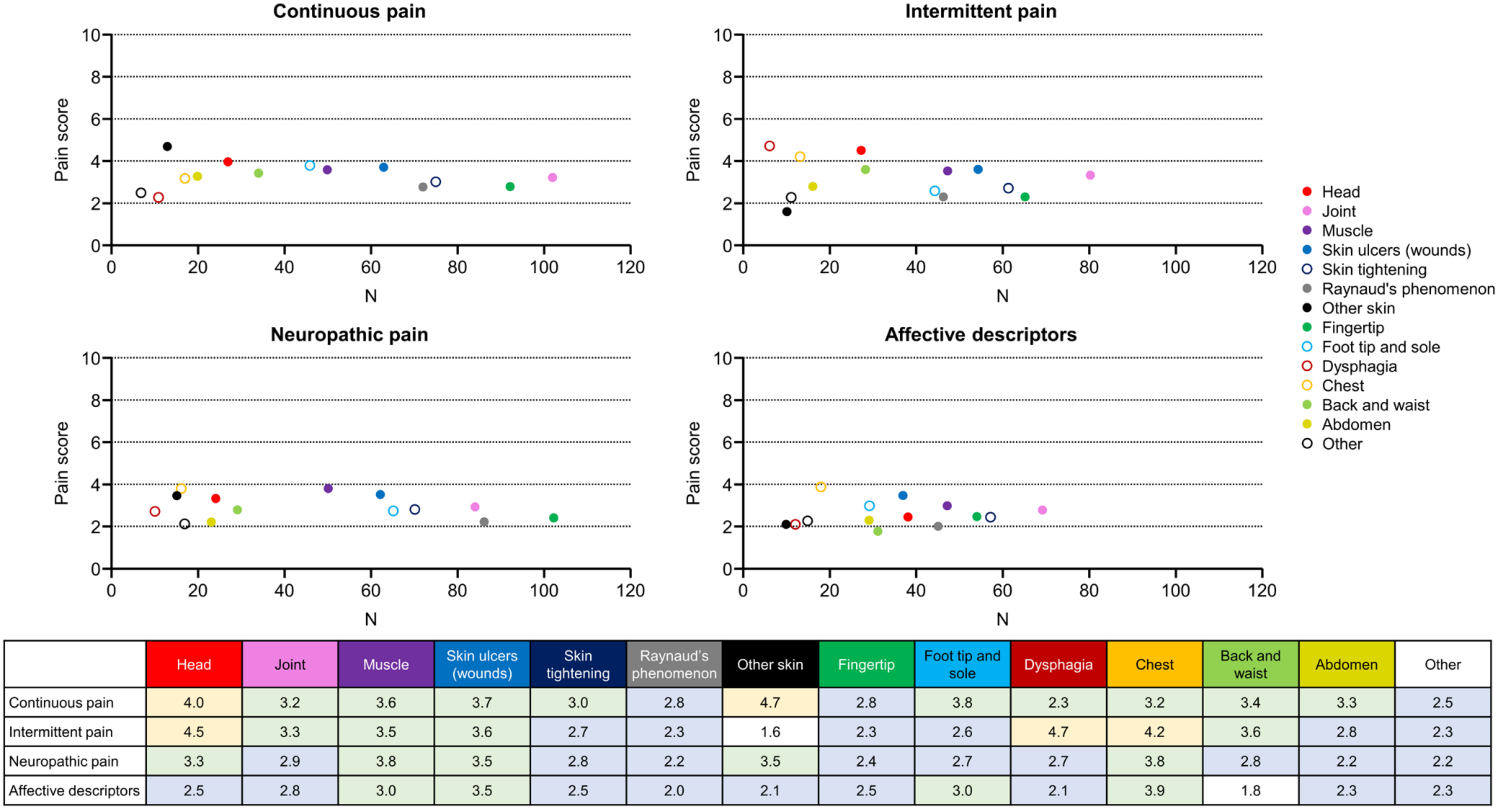
Scatter plot of the distribution of pain by type. The number of patients and median score for each pain location are shown for each type of pain. Each color indicates each pain location. The median pain scores are summarized in the table and color‐coded by score.

Modified SF‐MPQ‐2 scores by pain type, location, and prevalence are summarized in Figure 2. The highest median pain scores for continuous pain were for locations such as other skin (4.7), head (4.0), foot tip and sole (3.8), and skin ulcers (3.7). For intermittent pain, the highest median pain scores tended to be for dysphagia (4.7), head (4.5), chest (4.2), skin ulcers (3.6), back and waist (3.6), and muscle (3.5) pain. The median pain score for neuropathic pain was highest for patients with muscle and chest pain (3.8 each), followed by skin ulcers and other skin pain (3.5 each). The highest median scores for affective descriptors were for chest (3.9) and skin ulcers (3.5). Across all pain types, pain was most prevalent for joints, fingertips, RP, and skin tightening.

The prevalence of pain and pain scores were analyzed according to the SSc disease subtype (Table 1). More patients with lcSSc experienced pain, across the different pain types, in fingertips and locations affected by RP (RP and fingertips, respectively: continuous, 54 [42.9%] and 68 [54.0%]; intermittent 44 [41.1%] and 56 [52.3%]; neuropathic, 59 [44.0%] and 73 [54.5%]; affective descriptors, 44 [41.5%] and 54 [50.9%]) than patients with dsSSc (RP and fingertips, respectively: continuous, 37 [30.1%] and 47 [38.2%]; intermittent 33 [28.9%] and 44 [38.6%]; neuropathic, 42 [32.1%] and 51 [38.9%]; affective descriptors 36 [31.9%] and 42 [37.2%]). Conversely, more patients with dcSSc than with lcSSc experienced pain due to skin tightening (total 64 patients [48.1%] with dcSSc and 44 [32.1%] with lcSSc), skin ulcers (57 [42.9%] and 35 [25.5%]), muscle pain (52 [39.1%] and 39 [28.5%]), and back and waist pain (37 [27.8%] and 18 [13.1%]) (Table 1). Regarding pain severity, the median SF‐MPQ‐2 total scores were higher in most pain locations for patients with dcSSc, except in the “back and waist” area (Table 1).

**TABLE 1:** Patient locations of pain by limited (*n* = 143) or diffuse (*n* = 135) cutaneous systemic sclerosis or disease type classification.

	Pain classification, *n* (%)											
	Continuous		Intermittent		Neuropathic		Affective descriptors		Total		SF–MPQ–2 total score, median (Q1–Q3)	
Site of pain	lcSSc	dcSSc	lcSSc	dcSSc	lcSSc	dcSSc	lcSSc	dcSSc	lcSSc	dcSSc	lcSSc	dcSSc
Total	126 (100)	123 (100)	107 (100)	114 (100)	134 (100)	131 (100)	106 (100)	113 (100)	137	133	38.0 (12.0–75.0)	61.0 (20.0–104.0)
Head	27 (21.4)	30 (24.4)	25 (23.4)	28 (24.6)	27 (20.1)	30 (22.9)	26 (24.5)	28 (24.8)	27 (19.7)	30 (22.6)	63.0 (26.0–110.0)	83.5 (39.0–114.0)
Joint	67 (53.2)	66 (53.7)	59 (55.1)	61 (53.5)	71 (53.0)	66 (50.4)	59 (55.7)	62 (54.9)	72 (52.6)	67 (50.4)	50.0 (20.5–77.0)	78.0 (26.0–107.0)
**Muscle**	39 (31.0)	51 (41.5)	33 (30.8)	50 (43.9)	38 (28.4)	51 (38.9)	35 (33.0)	48 (42.5)	**39 (28.5)**	**52 (39.1)**	60.0 (24.0–97.0)	89.0 (50.0–117.0)
**Skin ulcers**	33 (26.2)	57 (46.3)	28 (26.2)	54 (47.4)	35 (26.1)	56 (42.7)	25 (23.6)	51 (45.1)	**35 (25.5)**	**57 (42.9)**	59.0 (19.0–77.0)	90.0 (48.0–112.0)
**Skin tightening**	42 (33.3)	61 (49.6)	38 (35.5)	56 (49.1)	42 (31.3)	64 (48.9)	35 (33.0)	58 (51.3)	**44 (32.1)**	**64 (48.1)**	45.5 (23.0–71.0)	67.5 (25.5–108.0)
**Raynaud's phenomenon**	54 (42.9)	37 (30.1)	44 (41.1)	33 (28.9)	59 (44.0)	42 (32.1)	44 (41.5)	36 (31.9)	**60 (43.8)**	**42 (31.6)**	26.5 (9.0–59.5)	68.0 (20.0–100.0)
Other skin	10 (7.9)	24 (19.5)	10 (9.3)	23 (20.2)	10 (7.5)	24 (18.3)	9 (8.5)	22 (19.5)	10 (7.3)	24 (18.0)	70.5 (43.0–101.0)	71.5 (35.0–100.5)
**Fingertip**	68 (54.0)	47 (38.2)	56 (52.3)	44 (38.6)	73 (54.5)	51 (38.9)	54 (50.9)	42 (37.2)	**74 (54.0)**	**51 (38.3)**	37.5 (13.0–74.0)	66.0 (20.0–100.0)
Foot tip and sole	41 (32.5)	35 (28.5)	31 (29.0)	32 (28.1)	44 (32.8)	38 (29.0)	34 (32.1)	31 (27.4)	45 (32.8)	38 (28.6)	38.0 (14.0–82.0)	66.0 (21.0–106.0)
Dysphagia	8 (6.3)	13 (10.6)	5 (4.7)	12 (10.5)	9 (6.7)	12 (9.2)	6 (5.7)	12 (10.6)	9 (6.6)	13 (9.8)	34.0 (25.0–101.0)	90.0 (66.0–122.0)
Chest	14 (11.1)	23 (18.7)	10 (9.3)	23 (20.2)	15 (11.2)	23 (17.6)	14 (13.2)	23 (20.4)	15 (10.9)	23 (17.3)	26.0 (12.0–66.0)	104.0 (61.0–137.0)
**Back and waist**	18 (14.3)	33 (26.8)	16 (15.0)	34 (29.8)	17 (12.7)	36 (27.5)	17 (16.0)	33 (29.2)	**18 (13.1)**	**37 (27.8)**	68.0 (17.0–113.0)	61.0 (20.0–106.0)
Abdomen	18 (14.3)	15 (12.2)	15 (14.0)	15 (13.2)	19 (14.2)	18 (13.7)	18 (17.0)	16 (14.2)	19 (13.9)	18 (13.5)	57.0 (26.0–84.0)	63.5 (20.0–111.0)
Other	12 (9.5)	11 (8.9)	12 (11.2)	10 (8.8)	13 (9.7)	12 (9.2)	10 (9.4)	7 (6.2)	13 (9.5)	12 (9.0)	37.0 (24.0–63.0)	45.0 (29.0–106.0)

*Note:* Bold font in table highlighted the site of pain and the total number of pain classification (%), where the difference between lcSSc and dcSSc exceeded 10%.

Abbreviations: dcSSc, diffuse cutaneous systemic sclerosis; lcSSc; limited cutaneous systemic sclerosis; Q, quartile; SF‐MPQ‐2, Short‐form McGill Pain Questionnaire.

**FIGURE 3:**
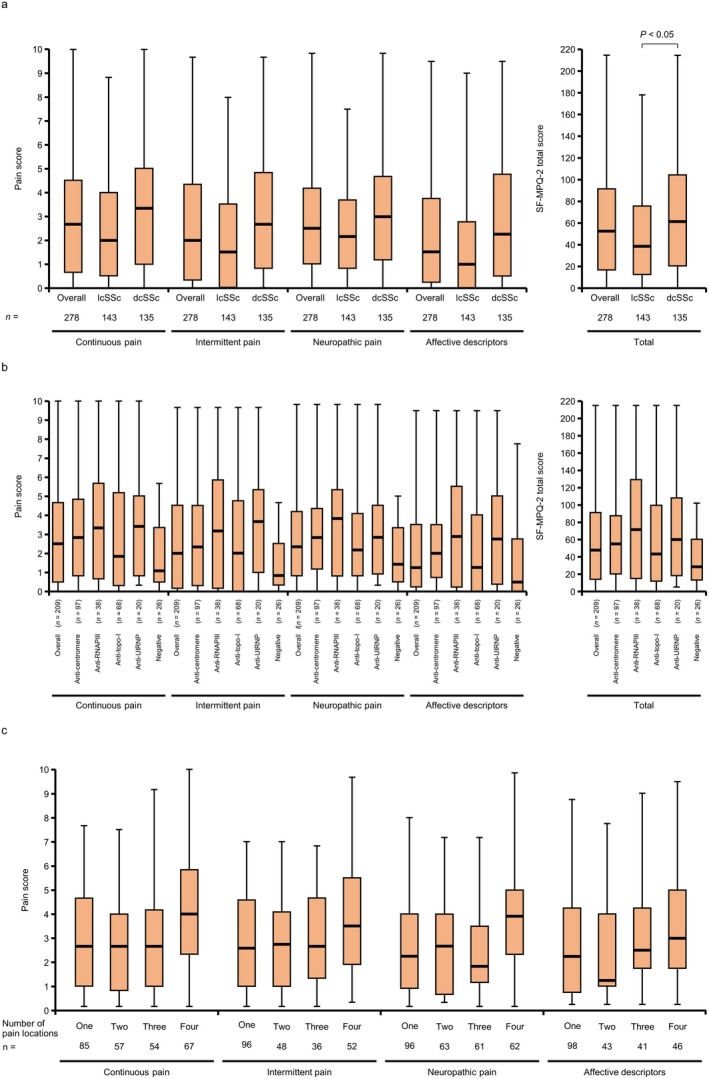
Pain scores across the four types of pain by (a) SSc subtype, (b) autoantibody status and (c) number of selected pain locations. The average of the SF‐MPQ‐2 questions applicable to each type of pain was calculated for each patient and used as the pain score. (a) Summary statistics were calculated for each patient's disease type. A Wilcoxon rank‐sum test was performed. (b) Summary statistics were calculated for each patient's autoantibodies. (c) Summary statistics were calculated for each patient's number of selected pain locations. The box represents quartiles 1–3, the line in the box shows the median value, and the upper and lower limits of the whiskers show the maximum and minimum values, respectively. SSc, systemic sclerosis; lcSSc, limited cutaneous systemic sclerosis; dcSSc, diffuse cutaneous systemic sclerosis; SF‐MPQ‐2, Short‐form McGill Pain Questionnaire; anti‐RNAPIII, anti‐RNA polymerase III; anti‐topo‐I, anti‐topoisomerase I. Overall: Anti‐centromere: Patients positive for anti‐centromere antibody. Anti‐RNAPIII: Patients positive for anti‐RNAPIII antibody. Anti‐topo‐I: Patients positive for anti‐topo‐I antibody. Anti‐U1RNP: Patients positive for anti‐U1RNP antibody. Negative: Patients negative for all the above antibodies.

Modified SF‐MPQ‐2 scores by pain type and SF‐MPQ‐2 total scores were compared by subgroup analyses. Patients with dcSSc had significantly higher pain scores than those with lcSSc (median [Q1–Q3]: continuous pain, lcSSc 2.0 [0.5–4.0] and dcSSc 3.3 [1.0–5.0]; intermittent pain, lcSSc 1.5 [0.0–3.5] and dcSSc 2.7 [0.8–4.8]; neuropathic pain, lcSSc 2.2 [0.8–3.7] and dcSSc 3.0 [1.2–4.7]; and affective descriptors, lcSSc 1.0 [0.0–2.8] and dcSSc 2.3 [0.5–4.8]) (Figure 3a). Similarly, patients positive for RNAPIII antibodies tended to have higher pain scores across the four types of pain (continuous pain, 3.3 [0.7–5.7]; intermittent pain, 3.2 [0.2–5.8]; neuropathic pain, 3.8 [0.8–5.3]; and affective descriptors, 2.9 [0.3–5.5]) (Figure 3b). Patients who selected the maximum number of pain locations, that is, four types of pain, tended to experience higher pain scores (continuous pain, 4.0 [2.3–5.8]; intermittent pain, 3.5 [1.9–5.5]; neuropathic pain, 3.9 [2.3–5.0]; and affective descriptors, 3.0 [1.8–5.0]) compared with those who experienced pain in 1–3 locations (Figure 3c). Patients with disease durations of 1–3 years had the greatest pain intensity, as evidenced by an SF‐MPQ‐2 total score of 58.0 (15.0–101.0), compared with 42.5 (17.0–83.5) for durations < 1 year, 40.5 (16.0–85.0) for 3–5 years, and 43.0 (11.0–78.0) for ≥ 5 years.

A subgroup analysis of the modified SF‐MPQ‐2 score by pain type and SF‐MPQ‐2 total score stratified by symptoms was performed (Table 2). The highest median (Q1–Q3) total SF‐MPQ‐2 pain scores were observed in patients who experienced nausea (69.0 [26.0–112.0]), insomnia (58.0 [13.0–109.0]), pain (58.0 [25.0–100.0]), diarrhea (57.5 [15.0–98.5]), and weakness (55.0 [19.0–92.0]).

**TABLE 2:** Patient symptom types and median pain scores.

Symptoms	*n*	Pain score median (*n*)				SF–MPQ–2 total score, median (Q1–Q3)
		Continuous	Intermittent	Neuropathic	Affective descriptors	
Nausea	47	3.7 (41)	3.7 (35)	3.7 (46)	3.3 (39)	69.0 (26.0–112.0)
Insomnia	57	3.7 (51)	3.4 (44)	3.3 (55)	2.5 (44)	58.0 (13.0–109.0)
Pain	106	3.3 (103)	2.8 (89)	3.0 (101)	2.5 (87)	58.0 (25.0–100.0)
Diarrhea	72	3.3 (63)	3.3 (53)	3.0 (70)	2.3 (58)	57.5 (15.0–98.5)
Weakness	109	3.2 (103)	3.3 (83)	3.0 (105)	2.5 (85)	55.0 (19.0–92.0)
Dysphagia	60	3.3 (57)	3.0 (46)	2.8 (58)	2.3 (47)	54.0 (17.0–96.0)
Palpitations	70	3.3 (62)	3.3 (53)	3.0 (67)	3.0 (52)	50.5 (14.0–109.0)
Skin ulcers	92	3.0 (85)	3.3 (73)	2.5 (92)	2.8 (71)	48.0 (16.5–101.0)
Joint contracture	137	2.8 (126)	2.8 (106)	2.7 (130)	2.3 (103)	48.0 (17.0–90.0)
Pyrexia	45	3.2 (43)	2.8 (39)	3.3 (42)	2.0 (39)	48.0 (23.0–92.0)
Skin tightening	190	2.9 (170)	2.8 (145)	2.5 (181)	2.3 (145)	47.0 (14.0–91.0)
Pigmentation and depigmentation	91	2.8 (81)	2.8 (72)	2.9 (86)	2.3 (70)	47.0 (20.0–90.0)
Malaise	145	2.8 (129)	2.6 (108)	2.8 (138)	2.3 (109)	44.0 (13.0–85.0)
Constipation	74	2.7 (67)	2.8 (54)	2.7 (69)	2.3 (57)	43.0 (16.0–90.0)
Dysarthria	43	2.8 (39)	3.0 (31)	2.5 (41)	2.8 (31)	42.0 (14.0–100.0)
Shortness of breath/difficulty breathing/cough	107	2.9 (92)	2.7 (77)	2.5 (101)	2.3 (76)	42.0 (12.0–87.0)
Swelling of fingers	184	2.7 (161)	2.7 (136)	2.3 (175)	2.0 (135)	37.0 (12.0–85.0)
Raynaud's phenomenon	153	2.5 (128)	2.3 (106)	2.3 (143)	2.0 (104)	33.0 (9.0–77.0)
Reflux esophagitis	121	2.2 (105)	2.2 (83)	2.3 (116)	2.0 (87)	32.0 (10.0–77.0)
Other	25	3.3 (17)	2.8 (16)	2.3 (21)	2.8 (15)	17.0 (4.0–76.0)

Abbreviation: SF‐MPQ‐2, Short‐form McGill Pain Questionnaire.

**FIGURE 4:**
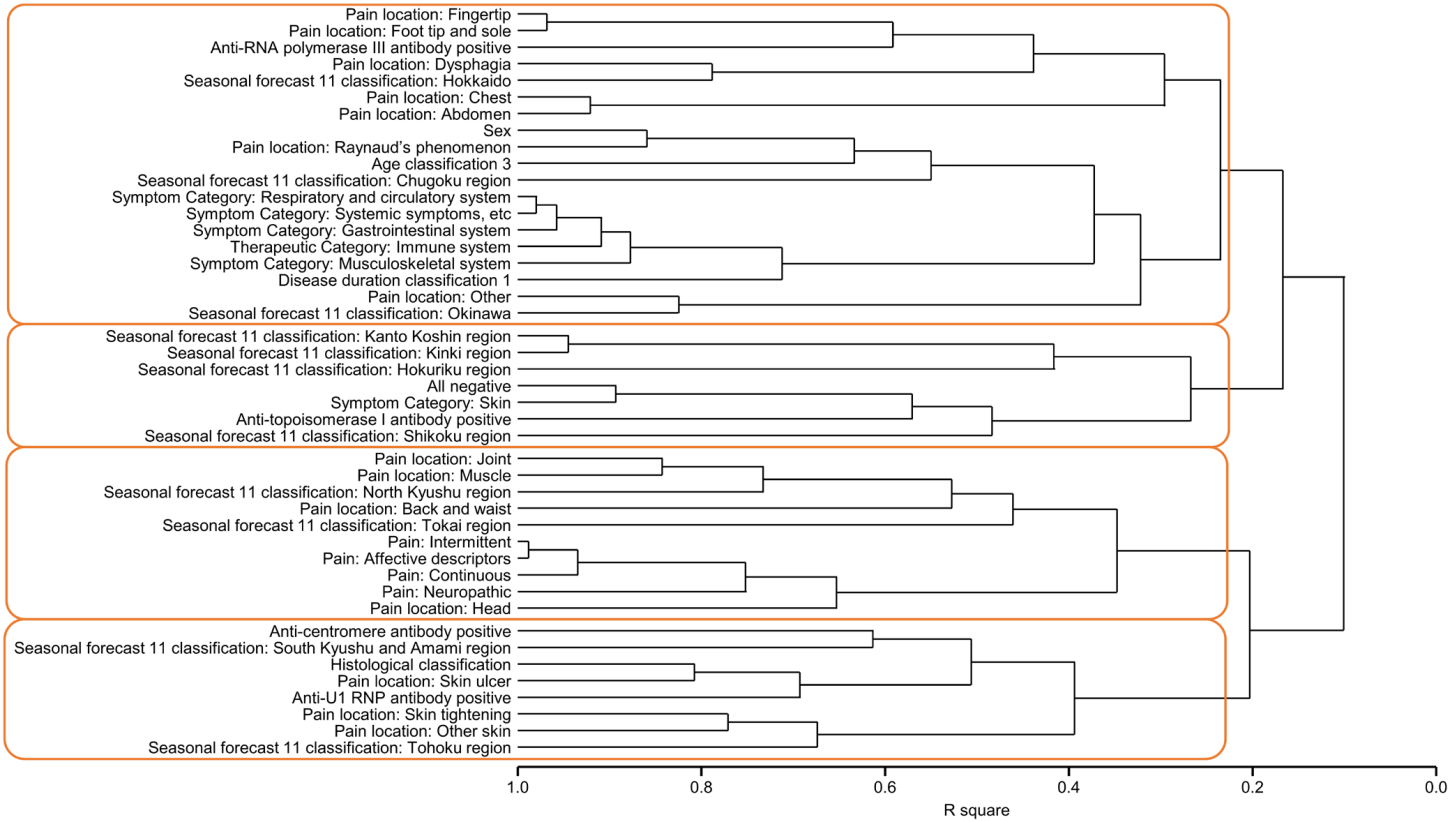
Relationship between pain location and patient background. According to patient backgrounds, the hierarchical clustering was performed, and factors were categorized into four groups. Anti‐RNAPIII, anti‐RNA polymerase III; anti‐topo‐I, anti‐topoisomerase I; R square, coefficient of determination.

Hierarchical clustering classified patients with SSc factors into four clusters (Figure 4). Although no associations were found in patient attributes or symptoms, some pain locations were clustered. The first cluster contained RP‐related or gastrointestinal symptom‐related pain, including foot tip and sole, chest, abdomen, RP, and ‘others’. The second cluster did not include any pain location, while the third cluster contained osteoarticular pain and headache, including joint, muscle, back and waist, and head pain. The fourth cluster contained skin tightening, skin ulcers, and other skin‐related pain.

The link between SSc symptoms experienced and pain location was analyzed using an estimated prevalence value. Higher scores indicate a stronger relationship between symptoms and pain location. Since the patients with skin ulcer symptoms and skin ulcer pain had a score of 1.90, a high relationship between symptoms and pain location was suggested if the score was greater than 2.00. Strong relationships were suggested between dysphagia symptoms and dysphagia pain (2.72) and dysarthria (2.04), between chest pain and palpitations (2.09), dysphagia (2.06), and diarrhea (2.04), between back and waist pain and pyrexia (2.01), and between abdomen pain and palpitations (2.00) (Table 3).

**TABLE 3:** Relationship between symptoms experienced and pain location.

Symptoms	*n*	Site of pain														Expected value (%)
		Head	Joint	Muscle	Skin ulcers (wounds)	Skin tightening	Raynaud's phenomenon	Other skin	Fingertip	Foot tip and sole	Dysphagia	Chest	Back and waist	Abdomen (stomach or abdominal pain)	Other	
Raynaud's phenomenon	153	1.11	1.08	1.06	1.02	0.77	1.43	0.87	1.21	1.19	1.15	1.16	1.02	1.10	1.21	50.8
Swelling of fingers	184	1.20	1.19	1.10	1.00	0.92	1.26	1.06	1.32	1.19	1.16	1.17	0.87	1.10	1.11	61.1
Skin tightening	190	1.08	1.05	0.93	1.12	1.31	1.12	0.93	1.15	1.12	1.19	1.02	0.82	0.88	1.18	63.1
Pigmentation and depigmentation	91	1.10	1.06	1.01	1.24	1.23	1.17	1.26	1.13	1.30	1.24	1.10	0.83	1.00	1.81	30.2
Skin ulcers	92	1.47	1.08	0.96	1.90	1.09	1.31	1.44	1.14	1.14	1.50	1.34	1.15	0.84	1.06	30.6
Joint contracture	137	1.21	1.35	1.11	0.97	1.08	1.10	1.16	1.38	1.26	1.19	1.24	1.03	1.18	1.20	45.5
Weakness	109	1.38	1.25	1.13	1.07	1.07	1.33	1.38	1.43	1.52	1.73	1.77	1.33	1.41	1.07	36.2
Reflux esophagitis	121	1.16	1.11	0.97	0.91	0.74	1.49	0.73	1.42	1.62	1.45	1.53	1.12	1.62	1.44	40.2
Diarrhea	72	1.11	1.17	1.19	1.30	0.93	1.33	1.72	1.36	1.55	1.39	2.04	1.46	1.75	0.94	23.9
Constipation	74	1.15	1.14	1.20	0.92	0.83	1.26	1.20	1.33	1.51	1.53	1.67	1.36	1.80	0.92	24.6
Nausea	47	1.81	1.23	1.01	1.38	1.07	1.16	1.13	1.23	1.30	1.60	1.97	1.39	1.79	1.24	15.6
Dysphagia	60	1.42	1.34	1.00	1.24	0.79	1.51	1.48	1.45	1.58	2.72	2.06	1.67	1.28	1.13	19.9
Dysarthria	43	1.28	1.44	0.88	1.13	0.71	1.08	1.03	1.40	1.65	2.04	0.90	1.28	0.65	1.58	14.3
Shortness of breath/difficulty breathing/cough	107	1.22	1.06	0.95	1.24	0.89	1.33	1.16	1.35	1.49	1.17	1.59	1.36	1.44	1.45	35.5
Palpitations	70	1.43	0.97	0.95	1.16	1.04	1.17	1.14	1.43	1.55	1.43	2.09	1.58	2.00	1.66	23.3
Malaise	145	1.21	1.09	1.07	1.03	1.00	1.25	1.22	1.21	1.31	1.21	1.65	1.35	1.45	1.41	48.2
Pyrexia	45	1.23	1.19	1.41	1.37	1.30	1.40	1.57	1.09	1.05	1.39	1.89	2.01	1.71	0.86	15.0
Insomnia	57	1.14	0.98	1.28	1.14	0.93	0.86	1.71	1.25	1.13	0.88	1.90	1.58	1.47	1.87	18.9
Pain	106	1.37	1.23	1.14	1.04	1.08	1.24	1.42	1.47	1.56	1.42	1.82	1.47	1.19	1.19	35.2
Other	25	0.80	0.99	0.63	0.39	0.33	0.77	1.42	0.89	0.81	0.50	1.85	0.80	1.96	1.94	8.3
Expected value (%)		19.9	48.5	31.6	30.9	35.9	36.5	11.3	44.9	29.6	8.0	13.0	19.9	14.3	10.3	

*Note:* Scores = (prevalence/301)/[(expected value of symptoms) × (expected value of pain location)].

The most common analgesic treatments prescribed to patients to manage pain regardless of pain location were steroids, non‐steroidal anti‐inflammatories/acetaminophen, and topical agents (57 [18.9%], 21 [7.0%], and 25 [8.3%] of 301 patients, respectively). Regarding the relationship between medications for the treatment of RP and headache, prostaglandins were prescribed to 38/301 patients (12.6%), of whom six (15.8%) had headaches (odds ratio [OR] 0.726 [95% confidence interval (CI): 0.289–1.825]); 18/301 patients (6.0%) were prescribed endothelin receptor antagonists, four (22.2%) of whom had headaches (OR 1.158 [95% CI: 0.367–3.654]); calcium channel blockers were prescribed to 17/301 patients (5.6%), three (17.6%) of whom had headaches (OR 0.853 [95% CI: 0.237–3.070]); and phosphodiesterase inhibitors were prescribed to 5/301 patients (1.7%), two (40.0%) of whom experienced headaches (OR 2.736 [95% CI: 0.447–16.751]).

When physicians were surveyed, 100/129 respondents estimated that, on average, 43.4% of patients experienced pain, which was less than that reported by patients. The other 29 physicians answered that they did not know how many patients experienced pain. Of the physicians surveyed, 76 (58.9%) prescribed painkillers to patients, 63 (48.8%) had asked patients to make efforts in their daily lives to alleviate pain, 55 (42.6%) believed that treating skin symptoms and skin ulcers could manage pain, and 21 (16.3%) had referred patients to a pain clinic or mental health care.

**FIGURE 5:**
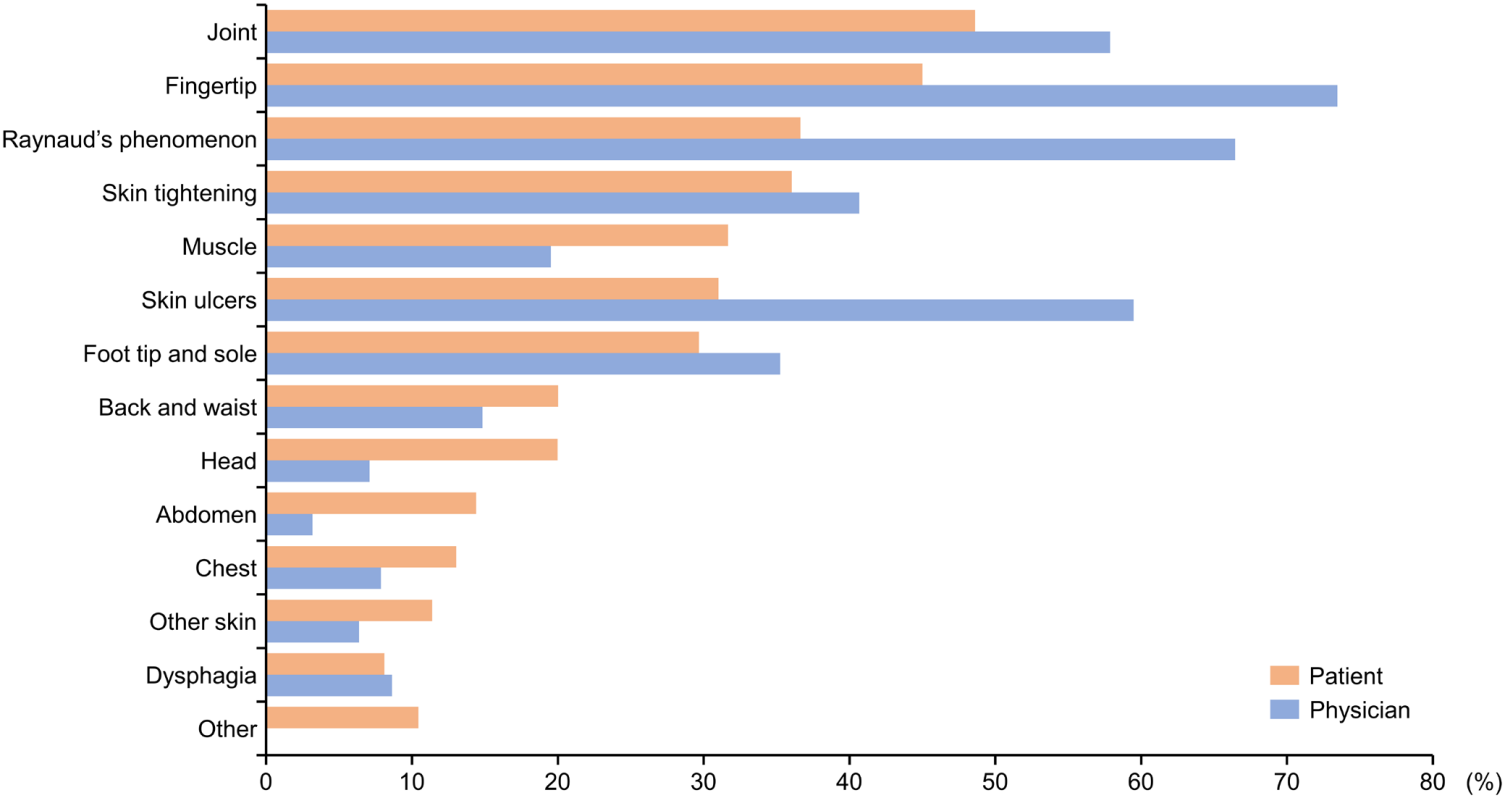
Most frequent pain locations by patient and physician perception. Patients: Percentages were calculated based on the site selected for any of the four types of pain. Physicians: Percentages were calculated based on the answers of which location of the body was thought to cause the pain.

Overall, the physicians believed that skin ulcers, RP, skin tightening, arthritis, and joint contractures were central to the symptoms causing pain (data not shown) and that the most common pain locations were the fingertips and locations affected by RP, skin ulcers, and joints (Figure 5). Physicians tended to be less aware of patients' commonly reported pain locations, such as muscle, head, and abdomen (Figure 5). Conversely, physicians perceived that more patients had pain associated with RP, skin ulcers, and fingertips than reported (Figure 5).

## 4 Discussion

To our knowledge, this is the first study to comprehensively analyze pain with SSc using the SF‐MPQ‐2. In this study, almost all (96.0%) patients complained of pain, which exceeded the physicians' perceptions of the number of patients experiencing pain (43.4%), highlighting a considerable gap in perception. Although patients reported that joints, fingertips, RP, and skin tightening were major pain locations, many physicians thought more patients would complain of pain from skin ulcers than skin tightening. Pain severity was highest in the throat, head, and chest. Cluster analysis showed a relationship between RP and gastrointestinal symptom pain, osteoarticular pain and headache, and skin tightening pain and skin ulcers. As for the relationship between symptoms and pain location, patients with chest pain were more likely to experience palpitations, dysphagia, and diarrhea. Similarly, there was a strong connection between dysphagia symptoms and dysphagia pain. Moreover, patients with nausea, insomnia, weakness, and diarrhea symptoms had higher pain scores.

In this study, continuous pain was the most common of the four types of pain. Continuous pain exhibited elevated scores among the commonly affected sites such as joints, fingertips, and areas with RP. A prior study similarly indicated that joint pain was persistent (78.6%) in SSc, followed by ischemic pain associated with RP (69%) [10]. Despite these symptoms fluctuating throughout the day, pain could be linked to ongoing tissue degeneration, such as joint destruction, occluded arteries, or ulcers, followed by continuous pain [32]. Continuous pain may need careful attention.

This study uncovered several connections between pain locations and symptoms. It is well established that reflux esophagitis can lead to chest pain [33]. Interestingly, in our investigation, we found strong associations between chest pain, nausea, and dysphagia, as well as reflux esophagitis, suggesting that undiagnosed reflux esophagitis could be a contributing factor. These reflux esophagitis‐related pain scores were high in this study, suggesting inadequate treatment, as in previous reports [34, 35, 36]. Additionally, our study revealed a notable association between chest pain and both diarrhea and constipation. Although the relationships between reflux esophagitis and gastrointestinal symptoms remain unclear, our results indicate a potential link between gastrointestinal symptoms and reflux esophagitis. We also found a link between pain associated with RP and gastrointestinal pain. It is possible that the side effects of therapeutic agents used to treat symptoms of RP, or vasculopathies similar to RP, may contribute to the development of gastrointestinal symptoms [37, 38, 39]. Indeed, gastrointestinal symptoms are present in many patients from the early stages [40], and a mechanistic and treatment link cannot be ruled out. Regarding medications and pain, a well‐established association between prostaglandin treatment and headaches is widely recognized [41]. Although their associations are well known [42], no clear association was found between drug treatments used for RP symptoms and headaches in the present study. This may be because patients who experienced headaches resulting from drug administration may have already stopped the medication. Although not directly demonstrated in this study, the locations in which pain is experienced somewhat imply associations with specific symptoms. Skin ulcers (e.g., digital ulcers) and RP are thought to occur concurrently in individual patients because of vasculopathy [43, 44], and ulcers at flexure sites are less frequently related to RP [45]. However, in this study, skin ulcer pain was more likely to be associated with pain from skin tightening rather than from RP. Skin ulcers exhibited a high pain score across all four pain types and generally pose challenges in terms of treatment and recovery. Thus, it is crucial to focus on preventive measures, such as treating skin tightening, to avoid the development of skin ulcers. Similarly, cluster analysis showed an association between “other skin” pain and skin ulcers, suggesting the skin at the flexion site may have been in a poor state before the formation of skin ulcers, which might constitute “other skin” pain. Importantly, the findings in this study suggest that the links between symptoms and pain locations were not necessarily direct associations. Further examination is necessary to demonstrate their associations.

In this study, patients with dcSSc had significantly more severe pain than those with lcSSc. This contrasts with previous research, which reported no significant difference in pain scores between the two subtypes [10]. This discrepancy may be due to differences in study design, patient characteristics, or the assessment tools used to measure pain. Psychosocial factors, comorbidities, or differences in treatment status across subgroups could have influenced pain perception and reporting. Further studies exploring these potential confounders are needed to clarify the relationship between SSc subtype and pain severity.

Moreover, patients with ≥ 4 pain locations and patients positive for anti‐RNAPIII antibodies had higher pain scores. Considering that the pain tends to be more severe at disease durations of 1–3 years, multiple comorbidities with high disease activity may lead to intense pain. Patients positive for anti‐RNAPIII antibodies are more prone to develop the dcSSc type of disease, characterized by rapid skin sclerosis, pigmentation, and pulmonary fibrosis [46]. In this study, 23/36 of the patients positive for anti‐RNAPIII antibody were diagnosed with dcSSc, and overall, they had more insomnia symptoms than patients positive for other antibodies in both the dcSSc and lcSSc groups. Insomnia has been reported to occur as a result of pain [47], further suggesting that patients with anti‐RNAPIII antibodies may experience more severe pain.

This study revealed a perception gap between patients and physicians. The actual number of patients reporting any pain was more than twice the number estimated by physicians. The findings indicate that it is unclear how well patients communicate their pain experiences to their physicians. This discrepancy may not only stem from physicians underestimating or failing to recognize pain, but also from patients feeling reluctant to, or finding it difficult to accurately, report their pain. Differences were also observed between physicians' perceptions and the actual distribution of pain locations. Furthermore, as noted in a previous report on this survey [27], it has been observed that patients often do not experience as much relief from their pain with treatment as physicians assume, and when patients do not feel any improvement in their pain, their treatment satisfaction decreases [27]. Therefore, physicians must be aware that their perception may differ from the actual situation and carefully evaluate pain from the patient's perspective.

The methodology of this survey has several limitations, as described previously [27]. First, there is a risk of selection bias. The findings presented here are based on the opinions of people who responded to our request, not a survey of all patients or a randomly selected sample. Second, there were differences in the nature of the responses given by doctors and patients. The patients responded based on their own experiences, whereas the physicians responded based on their general knowledge of the patient population. Third, there may have been a recall bias, as patients may have responded to strongly memorable items. Of note, patients were not matched to physicians who were seeing the patients; the inclusion of patients who had undergone or were undergoing various therapeutic interventions may have affected the results. A lack of comprehension of medical terminology may have confused the patients, and the responses made on behalf of patients may not have been accurate. Finally, the reliability and suitability of the Japanese version of the SF‐MPQ‐2 instrument for pain assessment have not been confirmed in the Japanese SSc population.

## 5 Conclusion

This study investigated the actual pain situation for patients with SSc and the differences in perception between patients and physicians. We found that the vast majority of patients with SSc experience pain, with the chest, head, and throat being the most painful sites, while the most common types of pain occur in joints, fingertips, sites affected by RP, and skin tightening. Moreover, we found important differences in the understanding of pain locations and perception between patients with SSc and physicians, and that listening to patients' experiences is essential for adequate and appropriate treatment of pain in SSc. Many patients with SSc experience pain and other symptoms, but physicians do not always recognize all of them, and thus, are unlikely to provide adequate treatment. The findings of this study make an important contribution to improving disease understanding and treatment satisfaction among patients with SSc.

## Ethics Statement

This study's protocol was approved by the Research Ethical Review Committee of Kyowa Kirin Co. Ltd. under the approval number EC_0105.

## Consent

Informed consent was obtained from all participants in this study and their representatives, where appropriate. The study adhered to the Declaration of Helsinki and the Ethical Guidelines for Medical Research Involving Human Subjects.

## Conflicts of Interest

Sei‐ichiro Motegi has received manuscript fees from Kyowa Kirin Co., Ltd.; grants or contracts from Taiho Pharmaceutical Co., Ltd., Sun Pharma Japan Ltd., Novartis Pharma K.K., Kaken Pharmaceutical Co., Ltd., Eli Lilly Japan K.K., and Maruho Co., Ltd.; and payment or honoraria from Janssen Pharmaceutical K.K., Eli Lilly Japan K.K., AbbVie G.K., Sanofi K.K., Daiichi Sankyo Co., Ltd., LEO Pharma K.K., Kyowa Kirin Co., Ltd., Nobelpharma Co., Ltd., Maruho, Co., Ltd., Torii Pharmaceutical Co., Ltd., Kaken Pharmaceutical Co., Ltd., Eisai Co., Ltd., Taiho Pharmaceutical Co., Ltd., Otsuka Pharmaceutical Co., Ltd., Bristol‐Myers Squibb K.K., and Sato Yakuhin Kogyo Co., Ltd. Yoshihito Shima has received manuscript fees from Kyowa Kirin Co., Ltd.; grants or contracts from Kobayashi Pharmaceutical Co., Ltd.; payment or honoraria from Asahi Kasei Pharma Corporation, Chugai Pharmaceutical Co., Ltd., and GlaxoSmithKline K.K.; and patents planned, issued, or pending from Kobayashi Pharmaceutical Co., Ltd. Mona Uchida‐Yamada, Taku Shimada, Haruka Ishii, Yoshito Ohya, and Yasumasa Kanai are employees of Kyowa Kirin Co., Ltd. Mona Uchida‐Yamada, Taku Shimada, Haruka Ishii, and Yoshito Ohya own stock in the company.

## Data Availability

Individual participant data, including data dictionaries, will be available for sharing. The shared data will encompass text, tables, figures, and appendices underlying the reported results after deidentification. Alongside this, documents such as the study protocol, reporting and analysis plan, and dataset specifications will also be accessible. The data will be available from the publication date until two years within that timeframe, and access will be granted to investigators with approved proposals by an independent review committee. Data will be made available through email correspondence with the corresponding author.
